# The impact of staffing on central venous catheter-associated bloodstream infections in preterm neonates – results of nation-wide cohort study in Germany

**DOI:** 10.1186/2047-2994-2-11

**Published:** 2013-04-04

**Authors:** Rasmus Leistner, Sarah Thürnagel, Frank Schwab, Brar Piening, Petra Gastmeier, Christine Geffers

**Affiliations:** 1 Institute of Hygiene and Environmental Medicine, German National Reference Center for the Surveillance of Healthcare-Associated Infections, Charité University Medicine Berlin, Hindenburgdamm 27, Berlin, Germany, 12203, Germany

**Keywords:** Staffing, CVC, BSI, NICU, VLBW

## Abstract

**Background:**

Very low birthweight (VLBW) newborns on neonatal intensive care units (NICU) are at increased risk for developing central venous catheter-associated bloodstream infections (CVC BSI). In addition to the established intrinsic risk factors of VLBW newborns, it is still not clear which process and structure parameters within NICUs influence the prevalence of CVC BSI.

**Methods:**

The study population consisted of VLBW newborns from NICUs that participated in the German nosocomial infection surveillance system for preterm infants (NEO-KISS) from January 2008 to June 2009. Structure and process parameters of NICUs were obtained by a questionnaire-based enquiry. Patient based date and the occurrence of BSI derived from the NEO-KISS database. The association between the requested parameters and the occurrance of CVC BSI and laboratory-confirmed BSI was analyzed by generalized estimating equations.

**Results:**

We analyzed data on 5,586 VLBW infants from 108 NICUs and found 954 BSI cases in 847 infants. Of all BSI cases, 414 (43%) were CVC-associated. The pooled incidence density of CVC BSI was 8.3 per 1,000 CVC days. The pooled CVC utilization ratio was 24.3 CVC-days per 100 patient days. A low realized staffing rate lead to an increased risk of CVC BSI (OR 1.47; p=0.008) and also of laboratory-confirmed CVC BSI (OR 1.78; p=0.028).

**Conclusions:**

Our findings show that low levels of realized staffing are associated with increased rates of CVC BSI on NICUs. Further studies are necessary to determine a threshold that should not be undercut.

## Background

Newborns with very low birthweight (VLBW) are at increased risk for developing healthcare-associated blood stream infections (BSI) [1-3]. The BSIs mostly occur on neonatal intensive care units (NICU) and are associated with the use of central venous catheters (CVC). To prevent these potentially lethal infections, consistent high quality of care is critical. Process and structure parameter on NICUs are suspected to influence the quality of care and therefore indirectly the incidence of CVC BSI. Until now it is not clear which parameters have the highest impact. Concerning this topic there have been only few studies on structure and process parameters on NICU that also include NICU staffing [4-6]. Analyzing data from the German nation-wide nosocomial infection surveillance system for preterm infants on neonatology departments and ICUs (NEO-KISS), we are able to estimate the situation for a large part of Germany’s NICUs.

## Methods

### Setting and patient population

In January 2000, NEO-KISS was started as a prospective national surveillance system for the most relevant nosocomial infections in VLBW infants (birthweight < 1500 g) in Germany [7]. By January 2008, 213 neonatology departments participated in KISS surveillance. In NEO-KISS, all VLBW infants admitted to the participating NICUs are kept under surveillance from admission to discharge, transfer or death, and all healthcare-associated infections are recorded.

### Data collection

We collected data on the structure characteristics of the neonatological departments and on the working processes of their NICUs with two separate point questionnaires. The first enquiry took place in August 2008, the second in April 2009. Information requested about NICU structure included data on number of wards and beds of the concerning departments, VLBW patient days in 2007, the existence of handover and/or infectious diseases rounds, and existence of microbiological screening, among other items. The NICU processes assessed included e.g. infusion preparation, CVC and incubator hygiene management, unit-level hand hygiene performance and infusion system use. The “realized staffing” for each unit was defined as realized number of nurses at the time of the questionnaire enquiry divided by the (individual hospital based) planned number of nurses. This ratio was multiplied by 100 and is displayed in the results section as “percentage of realized staffing”. Patient data was obtained from the NEO-KISS database for the departments that took part in our enquiry. The data from January 1^st^ 2008 to June 31^st^ 2009 were analyzed. In the NEO-KISS surveillance database, basic demographic data on patients (e.g. birthweight, sex, way of childbirth, device-days) is collected. Length of stay is defined as the number of days between birth or admission and until a weight of 1,800 g has been reached, the patient’s discharge or death. The pooled CVC utilization ratio was obtained as number of device-days per 100 patient-days. The pooled incidence density was defined as number of BSI cases per 1,000 patient days.

### Definitions of healthcare-associated infections

NEO-KISS focuses on bloodstream infections, pneumonia and necrotizing enterocolitis. Infection is classified as healthcare associated if arised in hospital after the first 72 h of life or 72 h after admission. NEO-KISS uses modified definitions from the Center for Disease Control and Prevention (CDC) for healthcare-associated infections. They are already thoroughly described in former publications [1,8]. A healthcare-associated BSI was considered central venous catheter-associated if the catheter was present within 48 hours before the infection occurred or if the catheter was still present at infection onset. According to the modified definitions of the CDC for primary sepsis, we stratified the cases in clinically-diagnosed BSI and laboratory-confirmed diagnosis (LCD) of BSI [1,8]. The LCD BSI cases were further classified by proven pathogens in the two groups coagulase negative staphylococci only (CoNS) or other than CoNS.

### Statistical analysis

In the descriptive analysis, we calculated numbers and percentages and/or median and inter-quartile range (IQR; 25% percentile - 75% percentile). In the multivariable analysis, we used logistic regression models to investigate the association between occurrence of CVC BSI and various patient, structure and process parameters. We investigated two different endpoints: CVC BSI and CVC BSI cases with laboratory confirmed (LCD) diagnosis other than CoNS. Since observations within one neonatology department are not statistically independent due to department-dependent policies, adjusted incidence rate ratios with 95% confidence intervals (CI) were estimated based on generalized estimating equation (GEE) models which account for this clustering effect by using an exchangeable correlation structure [9,10]. The log number of patient days was treated as an offset in the model. For the occurrence of bloodstream infection, the multivariable model building strategy was performed in 3 steps. First step: All patient-based parameters were considered in a logistic regression model by stepwise forward variable selection with the significance level p=0.09 for including a parameter in the model and p=0.10 for excluding a parameter. Second step: All process and structure parameters were considered in the resulting model from step 1 by stepwise forward variable selection using the same significance levels for including and excluding. Third step: With all parameters included by step 1 and 2, a GEE model was calculated that took cluster effects within a department into account. By stepwise backward selection only significant parameters remained in the final model. Excluding criteria were the smallest Chi-square value and p≥0.05 in the Type III score statistic. The quasi-likelihood information criterion (QIC) as a modification of the Akaike information criterion was used as goodness-of-fit measure in the GEE model. P-values less than 0.05 were considered significant. All analyses were performed using SAS (SAS Institute, Cary, NC, USA) [1-3]. In Germany, anonymised secondary data research does not require human research committee review.

## Results

### Basic characteristics of patients and departments

By January 2008, 213 neonatology departments were registered in NEO-KISS. Thereof 108 (51%) departments took part in our 2008 and 2009 inquiries and provided data on their VLBW-patients to the NEO-KISS database between January 2008 and June 2009 leading to a total of 5,586 patients with 206,459 patient days, and 50,113 CVC days. We found 954 BSIs that occurred in 847 (15.2%) infants. Table 1 shows the parameters of the participating NEO-KISS departments collected by the two questionnaires. Table 2 shows basic demographic data of the patients included. The distribution of realized staffing among the analyzed departments showed the 25% lowest achiever below a maximum level of 95% realized/planned staffing. This breakpoint was chosen as distinction between low level of staffing and not low level of staffing.

**Table 1:** Parameters of the analyzed NEO-KISS departments (N=108)

**Parameter**	**Category**	**N (%) / †median (IQR)**
Departments total		108 (100)
Size of department	Beds	†20 (16–29)
	ICU beds	†8 (6–12)
Patients in 2007	Total	†384 (298–529)
	< 1 500 g birthweight	†35 (20–55)
Patients January 2008- June 2009	< 1 500 g birthweight	†44 (28–65)
Level	Level III* (Neonatal critical care) departments	89 (82)
	Level II* (Step down neonatal nursery) departments	14 (13)
	Level I* (healthy baby nursery) departments	2 (2)
	other Level	3 (3)
Realized staffing percentage	Staffing	†99.6% (94.0% - 100.0%)
	Staffing (min-max)	87.0% - 117.0%
	Staffing ≥ 95%	72 (67)
	Staffing < 95%	27 (25)
	Missing	9 (8)
Daily handover rounds and/or regular infectious diseases rounds	No	2 (2)
	Yes	106 (98)
Standards for indication of hand hygiene	No	2 (2)
	Yes	104 (96)
	Missing	2 (2)
Daily disinfection of the buttons of the ventilation systems	No	6 (6)
	Yes	102 (94)
Routine microbiological screening	No	26 (24)
	Yes	82 (76)
Cleaning inside the incubator	No	25 (23)
	Yes	83 (77)
Infusion preparation	In the pharmacy OR on wards with laminar-flow bench	93 (86)
	On wards without laminar-flow bench	15 (14)

*Care level classification is based on the definitions by the National Healthcare Safety Network (NHSN).

IQR, inter-quartile range. Realized staffing percentage, ratio of realized staffing / planned staffing * 100.

**Table 2:** Basic patient characteristics of 5,586 analyzed VLBW neonates recorded in NEO-KISS between January 2008 and June 2009

**Parameter**	**N (%) overall**	**Median (IQR)**
VLBW neonates	5,586 (100)	
Sex male	2,836 (51)	
Birthweight in g		1,150 (869–1370)
Gestational age in days		205 (190–217)
Patient-days	206,459	33 (23–48)
Cesarean section	4,584 (82)	
Multiple birth	1,735 (31)	
VLBW neonates with ≥ 1 BSI	847 (15)	
CVC-days	50,113	5 (0–14)
1 CVC-associated BSI	349 (6)	
2 CVC-associated BSI	28 (1)	
3 CVC-associated BSI	3 (0)	
VLBW with ≥ 1 CVC-associated LCD BSI	94 (1.68)	
1 CVC-associated LCD BSI	89 (1.59)	
2 CVC-associated LCD BSI	4 (0.07)	
3 CVC-associated LCD BSI	1 (0.02)	
VLBW died before 1,800 g weight or discharge	358 (6)	
Realized staffing percentage		
Missing	551 (10)	
Staffing < 95%	1,403 (25)	
Staffing ≥ 95%	3,632 (65)	

IQR, inter-quartile range. VLBW, very low birthweight. CVC, central venous catheter. PVC, peripheral venous catheter. CPAP, continuous positive airway pressure. BSI, bloodstream infection. LCD, laboratory confirmed diagnosis. Realized staffing percentage, ratio of realized staffing/planned staffing * 100.

### Frequency of different BSI diagnoses

The pooled incidence of BSI was 15.2 per 100 patients, in median 11.7 (IQR 6.3-19.2). The pooled incidence density of BSI was 4.6 per 1000 patient days (median 3.5; IQR 2.1-5.7). Of the 954 (100%) cases of healthcare-associated BSI, 482 (49%) were laboratory confirmed. Two hundred fifty-eight cases (27%) were due to coagulase negative staphylococci only, 214 (22%) were due to pathogens other than CoNS. Four-hundred fourteen BSIs (43%) were CVC associated. The pooled incidence density was 8.3 per 1,000 CVC days (median 6.8, IQR 1.8-12.2). The pooled CVC utilization ratio was 24.3 per 100 patient days.

### Risk factors for CVC BSI

To assess the risk factors for a CVC BSI in VLBW newborns, we analyzed demographic, structure and process characteristics of their NICUs individually for each VLBW newborn. The results of the multivariable analyses are shown in Table 3. A realized staffing below 95% of planned staffing proved to be a significant risk factor for the development of CVC BSI compared to the reference category: realized staffing ≥ 95%.

**Table 3:** Risk factors associated with CVC-associated BSI on NEO-KISS NICUs

**Parameter**	**Category**	**OR**	**CI 95%**	**p-value**
Realized staffing percentage	Missing	1.25	(0.83-1.88)	0.289
	<95%	1.47	(1.11-1.95)	0.008
	≥95%	1=reference		
Birth weight	<500 g	4.23	(2.46-7.3)	<0.001
	500-749 g	3.17	(1.85-5.45)	<0.001
	750-999 g	2.28	(1.47-3.53)	<0.001
	1000-1249 g	1.36	(0.86-2.14)	0.187
	1250-1499 g	1=reference		
Gestational age (completed weeks)	<27 weeks	3.97	(2.23-7.09)	<0.001
	27-28 weeks	3.04	(1.79-5.16)	<0.001
	29-30 weeks	1.99	(1.05-3.77)	0.035
	>30 weeks	1=reference		
Length of stay	21-34 days	0.44	(0.29-0.69)	0.001
	35-48 days	0.32	(0.21-0.49)	<0.001
	>48 days	0.29	(0.2-0.42)	<0.001
	<21 days	1=reference		
Standards for indication of hand hygiene	Yes	0.61	(0.44-0.84)	0.002
	No	1=reference		
Daily disinfection of the buttons of the ventilation systems	Yes	0.68	(0.5-0.93)	0.014
	No	1=reference		
Disinfection of the application port before medication infusion/connection of an infusion system	Often	0.48	(0.31-0.77)	0.002
	Rarely/no	1=reference		
Infusion preparation	On ward without laminar-flow bench	1.53	(1.02-2.28)	0.039
	In the pharmacy OR on ward with laminar-flow bench	1=reference		

IQR, inter-quartile range. CI 95%, 95% confidence interval. CVC, central venous catheter. BSI, bloodstream infection. Realized staffing percentage, ratio of realized staffing / planned staffing * 100.

Results of the multivariable regression analysis by GEE models.

### Risk factors for LCD BSI other than CoNS

The multivariable analysis showed that low realized staffing is a risk factor for the development of laboratory confirmed CVC BSI due to organisms other than CoNS. The results are shown in Table 4.

**Table 4:** Risk factors associated with CVC-associated LCD BSI other than CoNS on NEO-KISS NICUs

**Parameter**	**Category**	**OR**	**CI 95%**	**P-value**
Realized staffing percentage	Missing	0.71	(0.26-1.94)	0.507
	<95%	1.78	(1.06-2.99)	0.028
	≥95%	1=reference		
Gestational age (completed weeks)	<27 weeks	10.73	(4.25-27.08)	<0.001
	27-28 weeks	2.72	(1.08-6.85)	0.033
	29-30 weeks	1.81	(0.67-4.88)	0.241
	>30 weeks	1=reference		
Mode of delivery	Vaginal	2.10	(1.29-3.43)	0.003
	Emergency cesarean section	1.05	(0.55-2.00)	0.888
	Cesarean section	1=reference		

CI 95%, 95% confidence interval. CVC, central venous catheter. BSI, bloodstream infection. LCD, laboratory confirmed diagnosis. CoNS, coagulase negative staphylococci. Realized staffing percentage, ratio of realized staffing / planned staffing * 100.

Results of the multivariable regression analysis by GEE models.

## Discussion

Multiple risk factors have an impact on the prevalence of CVC BSI in preterm neonates. We performed a prospective nation-wide study on the impact of process and structure parameters in the majority of German NICUs.

Our results demonstrate that high staffing levels are associated with a lower incidence of CVC BSI and laboratory-confirmed BSI (with organisms other than coagulase negative staphylococci). The results are congruent with several other studies on staffing [4,11]. A study by Pittet et al. demonstrated that an increased workload is associated with diminished hand hygiene compliance [12]. Cho et al. showed that even a small increase in the nurse-per-patient ratio is associated significantly decreased odds for adverse events [13]. Other studies showed that understaffing as well as overcrowding is associated with a higher risk of outbreaks on NICUs [14,15]. Two other studies did not observe an influence of nurse staffing [16,17]. However, both studies assessed the situation on ICUs rather than NICUs. We did not assess the nurse per patient ratio, but we showed that compliance with the in-house recommendations on staffing levels has the potential to prevent healthcare-associated BSI and is a relevant quality assurance tool. So far, there is no general reference for staffing ratios on NICUs. However, the German Commission on Hospital Hygiene and Infection Prevention suggests at least high levels of appropriately trained nurses [18].

Birthweight and gestational age have been shown in the literature to be the predominant patient related risk factors for healthcare-associated infections [2,19-21]. We could fully confirm these findings our study.

The preparation of infusions at laminar airflow benches has been recommended to minimize the risk of contamination [22,23]. Thomas et al. demonstrated that the training background of the preparing person can be critical, rather than the preparation site [24]. We did not assess the training background of personnel. Nevertheless, our data confirms the importance of laminar-flow benches in the preparation of intravenous fluids.

There are only few studies on the mode of delivery in VLBW newborns. It was found that cesarean section is associated with BSI in VLBW infants, while other studies reported improved morbidity and mortality [25-27]. Even though our results confirm the potential protection by cesarean section, this might also be an indirect effect of emergency vaginal delivery. Cesarean sections are usually handled in a well-prepared professional environment and more common in preterm infants [28].

This study has following limitations. It is based on the NEO-KISS database. Even though we thoroughly proofed their reliability, the accuracy of data is dependent on the quality of these data. We furthermore depend on the information we obtained by the inquiries that could be subject of relevant recall bias. We did not assess the nurse patient ratio and therefore cannot provide an exact threshold as benchmark for quality assurance. Nevertheless our results show that the planned numbers of staffing can have an impact on their rate of nosocomial infections. Planned staffing should therefore strictly be realized.

## Conclusions

We analyzed the impact of various process and structure parameters on the outcome of healthcare-associated BSI in VLBW neonates. A low level of staffing (realized/planned) on a NICU was associated with an increased risk for CVC-associated BSI. Rather high staffing levels should therefore be implemented and continuously be realized on NICUs. Our results furthermore emphasize the importance of standardization and consistency of medical procedures and hygiene measures on NICUs. We furthermore advocate a high level of communication and cooperation between the staff of the NICU and related fields like infectious diseases and clinical microbiology.

## Competing interests

The authors declare that they have no competing interests.

## Authors’ contributions

ST designed the questionnaires and organized the inquiry. BP organized the study and helped to draft the manuscript. RL, CG, FS and PG conceived of the study, participated in its design and coordination and drafted the manuscript. FS conducted the statistical analysis of the study. All authors read and approved the final manuscript.
